# AMX – the highly automated macromolecular crystallography (17-ID-1) beamline at the NSLS-II

**DOI:** 10.1107/S1600577522009377

**Published:** 2022-10-21

**Authors:** Dieter K. Schneider, Alexei S. Soares, Edwin O. Lazo, Dale F. Kreitler, Kun Qian, Martin R. Fuchs, Dileep K. Bhogadi, Steve Antonelli, Stuart S. Myers, Bruno S. Martins, John M. Skinner, Jun Aishima, Herbert J. Bernstein, Thomas Langdon, John Lara, Robert Petkus, Matt Cowan, Leonid Flaks, Thomas Smith, Grace Shea-McCarthy, Mourad Idir, Lei Huang, Oleg Chubar, Robert M. Sweet, Lonny E. Berman, Sean McSweeney, Jean Jakoncic

**Affiliations:** a New York Structural Biology Center, New York, New York, USA; b NSLS-II, Brookhaven National Laboratory, Upton, New York, USA; c LCLS, SLAC National Accelerator Laboratory, Menlo Park, California, USA; d Azure Data, Microsoft (United States), Atlanta, Georgia, USA; e Ronin Institute, Montclair, New Jersey, USA; f Cold Spring Harbor Laboratory, Cold Spring Harbor, New York, USA; g CSI, Brookhaven National Laboratory, Upton, New York, USA; h Physics Department, Brookhaven National Laboratory, Upton, New York, USA; Bhabha Atomic Research Centre, India

**Keywords:** macromolecular crystallography, automation, beamline, synchrotron source, high throughput, micro-beam, real-time feedback

## Abstract

The highly automated macromolecular crystallography beamline AMX/17-ID-1 is an undulator-based high-intensity (>5 × 10^12^ photons s^−1^), micro-focus (7 µm × 5 µm), low-divergence (1 mrad × 0.35 mrad) energy-tunable (5–18 keV) beamline at the NSLS-II, Brookhaven National Laboratory, Upton, NY, USA. It is one of the three life science beamlines constructed by the NIH under the ABBIX project and it shares sector 17-ID with the FMX beamline, the frontier micro-focus macromolecular crystallography beamline. AMX saw first light in March 2016 and started general user operation in February 2017. At AMX, emphasis has been placed on high throughput, high capacity, and automation to enable data collection from the most challenging projects using an intense micro-focus beam. Here, the current state and capabilities of the beamline are reported, and the different macromolecular crystallography experiments that are routinely performed at AMX/17-ID-1 as well as some plans for the near future are presented.

## Abstract 2

AMX (17-ID-1) is the highly automated macromolecular crystallography beamline at the NSLS-II. Photon delivery system, beamline instrumentation, high-performance computing environment and suites of applications are described for beamline scientists and users. AMX’s primary mission is to support routine structure determination from the most challenging projects.

## 1. Introduction

Macromolecular crystallography (MX), like other scientific fields, is in constant evolution benefiting in incremental improvements and transformative changes from technological developments in X-ray photon production, detection, high-precision positioning, parallel computing, and fast low-latency networks. Beam sizes are ever decreasing, beam fluxes are ever increasing, robots are getting faster and smaller, goniometer stages are getting smaller, faster and more precise, computers are getting more core counts/CPU, and data storage is getting denser and faster.

All these advances are required to enable data collection from smaller samples of various unpredictable qualities but more importantly to enable structural information from biological samples otherwise impossible to obtain. The level of automation in place in the latest state-of-the-art facilities together with the availability of automated data collection software, automated data processing and structure solution pipelines also enable high-throughput fragment screening and high-throughput ligand binding studies at extreme scales.

To this end, in 2013 the National Institute of Health (NIH) funded the construction of two dedicated MX beamlines and one life sciences scattering beamline at the NSLS-II: the highly Automated Macromolecular Crystallography (AMX), the Frontier micro-focus Macromolecular Crystallography (FMX) beamlines and the Life Science X-ray Scattering beamline (LIX) (Yang *et al.*, 2020 ▸). The NIH and the Department of Energy’s Office of Biological and Environmental Research (DOE BER) continuously fund the effort of these two MX beamlines to support researchers from academia and industry to perform experiments at AMX and FMX.

In the last year of operation, 2015, of the NSLS, six MX beamlines were in operation: two of them were undulator based and the rest used X-ray photons from bending magnet sources. The NSLS-II currently has three MX beamlines in operation: AMX (17-ID-1), FMX (17-ID-2) and NYX (19-ID; https://www.bnl.gov/nsls2/beamlines/beamline.php?r=19-ID). In the ten years since the beginning of these beamline projects, significant developments in X-ray sources, sample automation and high throughput for fragment screening as well as software have been implemented elsewhere. Most features were included, when possible, at the AMX and FMX beamlines. High-throughput fully automated data collection was implemented first at the MASSIF-1 beamline (Bowler *et al.*, 2015 ▸) at the European Synchrotron Radiation Facility (ESRF). It was further upgraded with the implementation of *MeshBest* (Melnikov *et al.*, 2018 ▸). Fragment screening at large scale is implemented at the Diamond Light Source (DLS) I04-1 beamline (Douangamath *et al.*, 2021 ▸). It benefits from developments at other DLS beamlines in unattended data collection and high-throughput fast automatic sample changers (O’Hea *et al.*, 2018 ▸). Development in automatic data collection from microcrystals was implemented at the SPring-8 MX beamlines under a project called *ZOO* (Hirata *et al.*, 2019 ▸). At AMX, we are aiming to implement the best of these features and collaborate with other facilities, when possible. An example is the series of High Data Rate MX (HDRMX: http://hdrmx.medsbio.org/) meetings involving detector, data processing and beamline scientists from facilities across the globe.

## 2. Beamline components

### 2.1. Photon delivery system

The NSLS-II is one of the newest, most advanced synchrotron light sources in the world; it is a medium-energy (3 GeV) synchrotron storage ring with a horizontal emittance of 0.8 nm rad (Smaluk *et al.*, 2019 ▸) that is achieved with a high-current electron beam of 400 mA with a ring circumference of 792 m. This low emittance together with the achievable mirror surface error quality makes a low-divergence beam with a sub-10 µm size possible. We achieved a beam size of 7 µm × 5 µm and a divergence of 1 mrad × 0.35 mrad; the beam characteristics are optimized to collect data from challenging MX projects including very large complexes. One of the challenges that was addressed was to build two independent high-performance MX beamlines with micro-focus beam on the same sector with only 2 mrad beam separation from the two independent in-vacuum undulators.

*Undulator.* AMX and FMX share a low-β short straight section in sector 17 of the NSLS-II storage ring. The upstream undulator (17-ID-1) is canted outboard by 1 mrad and delivers photons to the AMX line while the FMX line receives beam from the downstream undulator (17-ID-2) canted inboard by 1 mrad for a total canting angle of 2 mrad. The two undulators are identical by design and are 1.5 m-long in-vacuum undulators with a 21 mm period (IVU21) made of Hitachi NdFeB NEOMAX magnets. The minimum undulator gap under current operating conditions is 6.4 mm.

**Figure 1:**
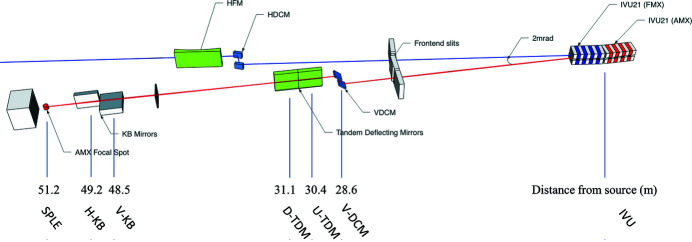
The AMX beamline layout (not to scale). AMX is served by the upstream (17-ID-1) IVU21 undulator, FMX by the downstream (17-ID-2) one. The beam of AMX is shown in red and that of FMX in blue. All distances are given with respect to the center of the short straight section, between the two undulators. The distance between the center of the AMX IVU and the center of the short straight section is 1.3 m.

*Optical layout.* The optical layout of AMX (Fuchs *et al.*, 2014 ▸, 2016 ▸; Berman *et al.*, 2011 ▸; Schneider *et al.*, 2013 ▸) is optimized to deliver a highly stable high-flux low-divergence beam into a 7 µm focus and to cover a photon energy range from 5 to 18 keV. These five characteristics must be achieved given the constraint that AMX shares one short straight section of the NSLS-II storage ring with its companion beamline FMX; therefore sufficient horizontal separation of the two independent beams is also required to accommodate instrumentation in the AMX endstation. The FMX photon delivery system has been described previously (Schneider *et al.*, 2021 ▸); here we focus on the AMX optical elements, which were fabricated by RI Research Instruments (GmbH, Germany). Three key elements were required to achieve the desired performance given the AMX/FMX beam separation constraint; a vertically deflecting double-crystal monochromator (DCM), followed by two identical flat deflecting monochromatic mirrors (hereafter referred to as tandem deflecting mirrors, TDM), and finally a Kirkpatrick–Baez (KB) bimorph mirror pair in the experimental endstation (17-ID-B hutch), 2 m upstream of the sample position (see Fig. 1 ▸).

#### 2.1.1. DCM

The first optical component is the vertically deflecting double-crystal monochromator (VDCM) that uses two flat Si(111) crystals to cover the 5–18 keV range. The horizontal Bragg rotation axis carries both crystals, with the first upstream crystal at the rotation center. The fixed exit beam geometry is achieved by translating the gap between the two crystals; the vertical upward offset is 30 mm. The companion beamline, FMX, relies on a horizontal deflecting DCM (HDCM) so that gravity-induced vibrations are minimized to enable a stable, low-vibration 1 µm vertical beam at the sample position, 27 m away from the HDCM. Furthermore, the horizontal orientation of the FMX DCM provides an additional 30 mm of separation between the two AMX/FMX beams. The Si(111) diffracted bandwidth is 1.3 × 10^−4^, or 1.75 eV, at the nominal AMX energy. The thermal heat load on the first crystal from the undulator radiation is managed by indirect liquid-nitro­gen (LN2) cooling of two clamped Cu heat exchangers. The LN2 then flows to the copper heat exchanger on top of the second crystal holder. Optionally, the second crystal holder can be indirectly cooled relying on copper braids between the two copper heat exchangers to further decouple vibrations. The current configuration used is the former: LN2 flowing between the copper heat exchangers of the first and second silicon crystal. The white beam of FMX passes through the VDCM vacuum chamber of the AMX DCM.

#### 2.1.2. TDM

To provide sufficient horizontal separation between the AMX and FMX branches and to implement higher harmonic rejection at energies below 8.5 keV, a pair of identical flat mirrors are the next optical components. Each mirror deflects the incoming beam 3.5 mrad outboard thus adding a 14 mrad deflection between the two beams. The tandem deflections provide sufficient separation 20 m downstream at the AMX sample position to allow development of a novel inhouse goniometer. Note that there is additional further separation provided by the HDCM at FMX with 30 mm extra on the inboard side and that the deflection between the two IVUs, 2 mrad, provides a total separation of 450 mm at the AMX sample position. Both TDMs (ZEISS), which are identical, have a 500 mm-long active area comprising a bare Si active stripe and a coated Pd active stripe, both 10 mm in width. Switching between the two stripes is automatic depending on the energy and takes less than 30 s. The measured RMS tangential slope error surface over the length of the mirrors (500 mm) is 0.18 and 0.19 µrad RMS for the two mirrors.

#### 2.1.3. KB mirrors

Focusing at the sample position at AMX is achieved by a pair of KB mirrors in the vertical and then the horizontal directions both at incidence angles of 3.5 mrad. The two mirrors are pre-shaped, to a meridional cylinder for the vertical mirror and to a meridional ellipse for the horizontal mirror. They are made of fused silica substrate with one Pd active stripe 600 mm long and 10 mm wide. They are second-generation bimorph mirrors with 16 side-attached piezo bending elements each (SESO NewGen) to achieve the final meridional ellipse shape for both mirrors. The measured tangential slope errors of both the H- and V-KB bent mirrors are 0.2 µrad RMS and 0.3 µrad RMS, respectively. Measurements were made in the NSLS-II dedicated metrology laboratory using the stitching Shack–Hartmann method. The working distance (and demagnification) of the V- and H-KB mirrors are 2680 (19) and 1970 (25) mm, respectively. With these specifications, the divergence of the focused beam at AMX is 1 mrad × 0.35 mrad, optimized to collect data from large complexes without the need to further control divergence using slits upstream of the focusing optics. Given the pre-shaping of the mirrors and the range available from the 16 piezo binding elements, the working distance ranges for the vertical focusing is 2600–4000 mm and for the horizontal focusing is 2000–4000 mm.

For each mirror, voltages are supplied to the 16 piezo elements of each bimorph using 8-channel high-voltage bipolar power supplies manufactured by Spellman (Hauppauge, NY, USA). They are repackaged and customized by FMB-Oxford/RI in rack mount units containing either 16 or 32 channels, depending on the numbers of piezo elements and mirrors. In the case of AMX, one rack unit is used for the two KB mirrors. A web application is provided to safely operate the bimorph following well defined boundary conditions (±2000 V maximum on each electrode and ±500 V between adjacent piezo elements). An Experimental Physics and Industrial Control System (EPICS) input/output controller (IOC) is available too to operate the bimorphs. Individual electrodes of the bimorph mirror can be set with a 0.1 V resolution.

#### 2.1.4. Focusing/achievable beam size/measured flux

The designed focusing scheme at AMX relies solely on the bimorph mirror surface shape control to achieve the smallest beam size at the sample and larger beam sizes moving the focal spot downstream of the sample up to about 1500–2000 mm from the sample. Beam-size control appears an attractive option to rapidly adapt the beam size to the current needs depending on the sample under investigation. One can rapidly expand the beam to probe very large samples or to rapidly interrogate crystal drops in *in situ* trays. Once samples are found, the beam can be re-focused to a desired size so that the beam size optimally matches the sample size, for maximal signal-to-noise ratio, reducing scattering from surrounding volumes. However, with the fast rastering available at AMX and the frequent vector data collections, the beam change feature is not used during daily routine operation. As a result, the beam size of AMX, although possible to change from approximately 7 to 100 µm, is maintained focused to a measured beam size of 7 µm × 5 µm. The AMX endstation instrumentation is designed to accept the focused beam and the unfocused beam, where the two KB mirrors are fully retracted so that the ∼2800 µm × 2300 µm low-divergence beam can be used to study samples with larger cell parameters. In this configuration, the beam flux through a 50 µm × 50 µm aperture would be 2 × 10^9^ photons s^−1^. A reduction in flux density from 1.3 × 10^11^ photons s^−1^ µm^−1^ to 8.1 × 10^6^ photons s^−1^ µm^−1^, a reduction of 1.6 × 10^4^-fold, nevertheless provides sufficient flux to collect data from large samples.

AMX currently delivers one beam size, consistently measured at 7 µm × 5 µm throughout the energy range 5–18 keV. This beam size is optimized for the vast majority of samples tested at the beamline and only a handful of requests have been made to deliver a larger beam and in one case a smaller beam. However, we are currently optimizing a second beam size of 25 µm × 25 µm to be used for data collection at room temperature.

**Figure 2:**
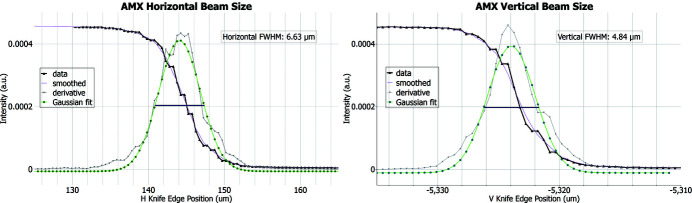
AMX beam profile obtained during the hot commissioning of the beamline (2016). AMX beam spot at focus as detected by a tungsten knife-edge scan in the horizontal and vertical directions. Left: horizontal profile with a 6.6 µm FWHM. Right: vertical profile with a 4.8 µm FWHM.

The beam size is optimized controlling the mirror figure of the two KB mirrors [see description by Schneider *et al.* (2021 ▸)]. The 16 electrode voltages are optimized and remain optimized for a long period of time, only requiring small manual tweaks globally applied to all electrodes from each mirror. The first round of such *in situ* optimization was conducted during the hot commissioning of the AMX beamline in the fall of 2016. The pencil-beam scanning method [fly-scan using *Bluesky* (Allan *et al.*, 2019 ▸) in *Jupyter Notebook*], following methods from Sutter *et al.* (2013 ▸), was used to optimize voltages from the 16 electrodes on each mirror. The measurement optimization cycles were repeated until the beam size converged to a measured beam size of 6.6 µm (H) × 4.8 µm (V) FWHM (see Fig. 2 ▸.). Beam size at the sample position is routinely measured using two methods. Method 1 relies on imaging the beam on either a YAG:Ce or a CdWO_4_ polished thin crystal in the focal plane with the attenuated beam; the beam image is then captured on the high-resolution on-axis microscope and analyzed to determine the FWHM in both the horizontal and the vertical directions. Method 2 relies on a knife-edge tungsten blade scan performed at the AMX focal spot in the horizontal and the vertical directions. Based on our experience, the knife-edge scan and the imaging method give very similar results with a discrepancy below 1 µm.

**Figure 3:**
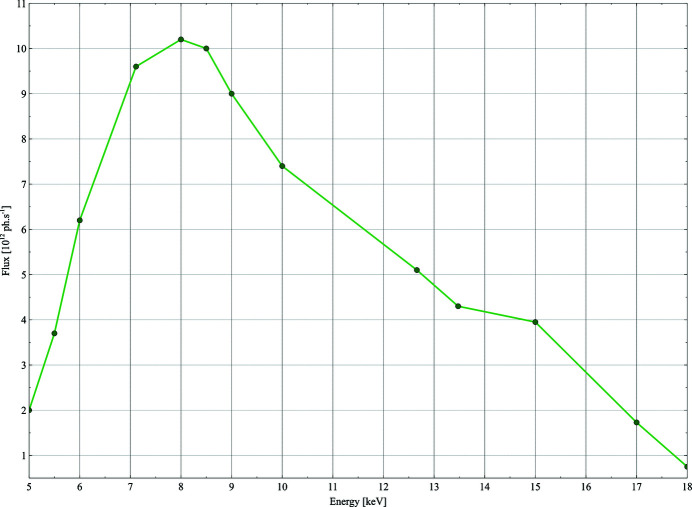
AMX photon flux at the sample position across the photon energy range 5–18 keV. Measurements performed at 400 mA ring current. At lower energies, the flux is diminished due to absorption in the Be exit window and the remaining air path to the sample (∼30 cm). A 50 µm-thick electronic-grade CVD diamond single-crystal BPM further attenuates the beam intensity but can be retracted.

The beam flux is measured routinely (see Fig. 3 ▸) using a 300 µm Si PIN photodiode from Hamamatstu. The flux is used as an input for a simple calculation to estimate the lifetime of a crystal of an average protein (250 residues, *a* = *b* = *c* = 125 Å, α = β = γ = 90°, and six copies per asymmetric unit cell) based on the size of the beam of an average protein; this information is made available to users on the data acquisition GUI.

The calculation of the lifetime is a simple estimation assuming a crystal sample with horizontal and vertical dimensions of 7 µm × 5 µm and thickness 25 µm in the third dimension, along the beam. With these parameters, we used *RADDOSE* (Paithankar & Garman, 2010 ▸) to calculate the dose (in MGy) per 10^12^ photons at every energy between 5 and 18 keV in 1 keV steps and extrapolated the dose for all values. The flux of the beamline (with no attenuators), measured at least twice a day or when an energy change greater than 25 eV is requested, is made available along with the current attenuation. These parameters are then used to compute in real time the ‘lifetime’ of that average sample. Users are made aware that this is a qualitative parameter to be used with caution but not to ignore. Using the improved automated data collection workflow (see description in Section 4.5), we are planning to estimate the sample size in three dimensions and use that information together with the flux, the transmission and the energy to calculate on-the-fly the dose for a standard data collection and a vector data collection using a local copy of the *RADDOSE-3D* API (Bury *et al.*, 2018 ▸).

#### 2.1.5. Diagnostics

For an extensive description of devices used for diagnostics and feedback including diamond screen, YAG:Ce insertable screens, chemical vapor deposition (CVD) diamond screen, beam-position monitor (BPM) and sets of scannable slits, and fast encoder readout FFT real-time measurements, see Schneider *et al.* (2021 ▸). AMX has one white-beam insertable CVD diamond visualization screen upstream of the DCM, insertable YAG screen and scannable slits before every optical element plus two pairs of slits near the sample position, the dynamics slits and the beam condition unit (BCU) slits. Finally, AMX employs the above-described 300 µm Si photodiode 25 mm downstream of the sample for beamline optimization and routine flux measurement as well as a CdWO_4_ screen at the sample location for manual or automated beam location correction. The most relevant beamline parameters are shown in Table 1 ▸.

Like FMX, AMX employs a quad-electrode CVD diamond BPM (Sydor Instruments SI-DBPM-M405) (Keister *et al.*, 2018 ▸) in the BCU approximately 20 cm upstream of the sample. For more details, see Section 2.2 and Schneider *et al.* (2021 ▸). The BPM can be retracted to decrease absorption at low energies (6 keV and below). The BCU is flushed with nitro­gen gas to protect the electrodes, but the flux densities at the beamline are high enough to cause damage under continuous exposure. Therefore, the beam position is regularly checked and corrected for drifts, with the BPM retracted while not in use. One key contributor to drifts is variation of the electron orbit, which is corrected in an accelerator-based feedback to a front-end BPM. At AMX, we found that the beam position at the sample depends mostly on the temperature of the endstation where the KB mirrors are located. We have implemented temperature stabilization within ±0.1° to improve beam stability at the sample position, where initial results indicate drifts smaller than 1 µm between two user start-ups (3 pm and 8 am the next day).

### 2.2. Experimental endstation

**Figure 4:**
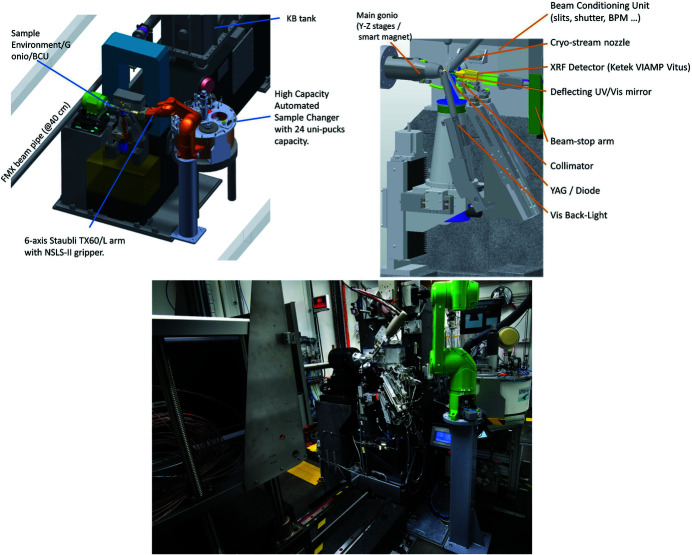
AMX endstation. Top left: design of the sample environment including most devices. Top-right: design of the end-station showing the main components. Bottom: photograph of the AMX endstation with the Eiger X 9M on the left and the six-axis TX60L arm/24-unipucks dewar on the right.

The available space of 400 mm between the AMX beam focal spot and the FMX beam flight path in the AMX endstation, together with the short working distance of 190 mm between the H-KB mirror of FMX and the sample position at FMX, dictated a novel design of the diffractometer (Fig. 4 ▸). The required elements of the experimental endstation including the main goniometer, used for data collection from crystals in standard SPINE bases (Cipriani *et al.*, 2006 ▸) at 100 K, the high-magnification high-resolution on-axis microscope, the BCU and all other required instrumentations to deliver a stable micro-focus beam, were all designed to be as identical as possible for both AMX and FMX. These instruments are extensively described in the FMX article (Schneider *et al.*, 2021 ▸). The main differences are described here: AMX is designed to perform high-throughput MX experiments on cryo-cooled samples and on a compact high-density *in situ* inhouse-developed crystallization tray (Soares *et al.*, 2021 ▸); as a result, AMX relies on one high-resolution (<1 µm measured sphere of confusion at the sample position) high-speed goniometer, identical to the main goniometer of FMX with the exception that one of the sample stages (PinY) offers longer travel so that the inhouse room-temperature (RT) compact tray can be scanned using standard rastering stages (Gonio-X, PinY and PinZ). The other noticeable difference is related to the longer translation required to collect data on samples necessitating the unfocused beam at AMX; to this end, an XY lift wedge was implemented so that the BCU plus microscope and all associated devices (beam stop, collimator, back light, front light, XRF detector) can follow the two main beam positions (focused and unfocused) that are 14 mm (H) × 19 mm (V) apart. To further reduce the air scatter and background, we implemented a smaller collimator, beamstop and drilled on-axis microscope deflection mirror. The collimator is a 25 mm-long platinum tube, 5 mm upstream of the sample with an internal diameter of 160 µm. The brass end-cap piece of the collimator is located 100 µm away from the deflection mirror that has a 1.2 mm centered drilled hole letting the focused beam pass through while offering ‘always on’ high-resolution on-axis sample visualization. The beamstop is located 9.5 mm downstream of the sample and is made of tungsten machined by wire erosion with a diameter of 400 µm.

#### 2.2.1. Detector

During the technical and scientific commissioning of the beamline (March 2016 to December 2016) a Dectris Pilatus 6M, originally from the X25 beamline (Héroux *et al.*, 2014 ▸) at the NSLS, was in place. The Pilatus 6M was upgraded to operate at 25 Hz. The current detector in use at AMX is a hybrid pixel-array single-photon-counting detector from Dectris, Eiger X 9M, with pixel size of 75 µm × 75 µm, a total number of 3110 × 3269 (10.2 M) pixels and an active area of 233 mm × 245 mm. The Eiger X 9M detector has a maximum frame rate of 238 Hz in the full area mode (9M) and can be triggered in the 4M ROI at up to 750 Hz. The detector is operated in file writer mode, where the detector control units write hdf5 data files along with the hdf5 master file containing all necessary metadata (Bernstein *et al.*, 2020 ▸) to the NSLS-II central computing facility over a combination of 10 and 40 Gb network links to a dedicated SSD buffer array with 16 TB capacity. Users routinely take advantage of the Eiger X 9M high framing rate of 200 Hz collecting either rotation data or rastering data with 5 ms exposure time. We plan to use the 4M ROI mode running at 750 Hz for specialized experiments including screening of crystallization conditions in our inhouse RT mini-tray (Soares *et al.*, 2021 ▸) and for our *rasterScreen* protocol (see Section 2.4.1 for the description).

The detector support assembly has three motorized stages – vertical, horizontal and along the beam – and the minimum detector distance is 100 mm. For data collection at long wavelengths, below 7 keV, we are planning to implement a helium flight path for a fixed detector distance of 100 mm following the design implemented at the FMX beamline (Karasawa *et al.*, in preparation), which will significantly minimize absorption of the radiation diffracted from the crystal and thereby increase the signal-to-noise ratio.

#### 2.2.2. Sample automation

AMX and FMX both use identical sample automation systems except that AMX relies on the extended version of the Staubli TX60 robotic arm, the TX60-L. The systems consist of a customized commercial automated sample storage cryo-Dewar (Absolut Systems) based on ESRF developments for the MASSIF-3 beamline (von Stetten *et al.*, 2020 ▸) that can hold up to 384 samples in 24 Uni-pucks. The six-axis robotic arm is outfitted with an inhouse gripper based on an original design from the Advanced Light Source, USA. The gripper is significantly modified and functionalized with several sensors (Lazo *et al.*, 2021 ▸) including a force torque real time sensor. Machine vision applications detect the presence and position of the sample holder in the gripper and the goniometer. We currently achieve a 35 s sample exchange time defined as the time it takes for a sample to be removed and the next sample to be mounted. During these sample exchanges the temperature, the state of the endstation, and the location of the pin are checked at the crucial steps to guarantee safe sample exchange. The current reliability of the sample exchange is greater than 99.9%, *i.e.* we do lose about one sample for every 1000 samples mounted. Reliability is key for AMX operation – it is achieved by accepting only SPINE pins (Cipriani *et al.*, 2006 ▸) in Uni-pucks. The high-precision goniometer requires the SPINE precision. The gripper has a built-in temperature sensor near the crystal location, and the high-capacity sample Dewar has a dedicated ‘cold park’ port allowing the gripper to stay cold (<−180°C) during sample collection and for days with no frost formation or gripper malfunction.

**Figure 5:**
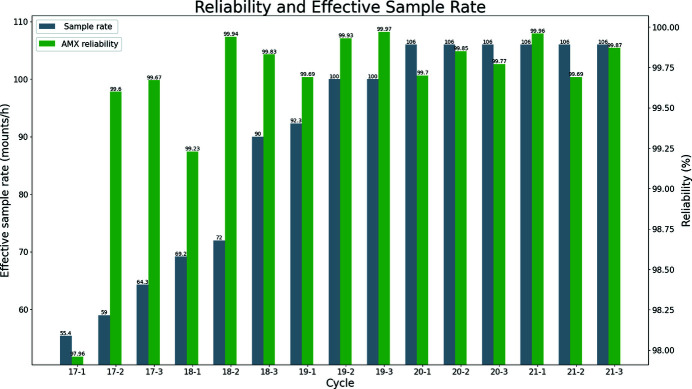
The sample rate (gray) is the number of samples the robot can mount excluding all other procedures such as crystal centering or data collection. Green represents reliability expressed as the percentage of successfully mounted samples. For a detailed definition of reliability, see Lazo *et al.* (2021 ▸).

The Dewar and ‘robot’ were installed at AMX in January 2017 and were made available for user operation in March 2017 on cycle 2017-2. Sustained continuous progress in reliability, efficiency and speed has been achieved, highlighted in the number of samples, effective sample exchange speed and reliability shown in Fig. 5 ▸.

We are now working on the next-generation gripper and a ‘floating lid’ to upgrade the pneumatic actuated lid to further increase throughput and sample exchange speed while increasing reliability. The force torque sensor feedback is routinely used to detect collision and stop the robot motion within 50 ms. It is also used for fine-tuning teachable positions. Additionally, each MX beamline is fitted with three proximity sensors (Eddy current from Lion Precision) for routinely validating the gripper position and when swapping grippers. Each beamline has two calibrated grippers that can be manually swapped. The current gripper has mounted more than 30000 samples with no required re-work.

### 2.3. Computing infrastructure

#### 2.3.1. Computing/network infrastructures

In order to achieve near-real-time feedback from rastering and automated data reduction, from data collected on the Dectris Eiger X 9M detector, data are written to the NSLS-II central computing facility, located about 500 m away from the beamline. Network connection speeds from the detector data collection unit (DCU) and the central storage range from 10 Gbps to 56 Gbps. All data processing compute nodes are also located in the NSLS-II central computing facility, and they are all connected to the General Purpose File System (GPFS) with 56 Gbps InfiniBand. Raw and processed data are written onto NSLS-II GPFS. We use two tiers of storage, both on GPFS. Firstly, a fast buffer storage made of high-speed/high-IO enterprise SSD drives in RAID10 with 18 TB of available storage. It is configured for writing and reading data only, in the form of hdf5 and cbf files. The fast buffer storage can store up to one day of the most intense data collection experiments, and each of the two MX beamlines AMX/FMX has a dedicated buffer that can be accessed by the other beamline. When either the buffer reaches a predetermined threshold (75% full) or files reach an age (set as 24 h), cbf and hdf5 files are transferred to a 1 PB GPFS medium-/long-term storage that is built upon spinning drive appliances. All temporary files, all non-cbf, non-hdf5 files are written to the 1 PB GPFS. In case of a catastrophic event such as loss of network, the beamline has a high-performance 4 TB PCI-X SSD. This 4 TB drive is NFS exported to all relevant compute nodes (in the NSLS-II controls), workstations and servers at the beamline. It provides limited but continuous operation for up to one day.

#### 2.3.2. Data processing

*Raster/spot finder.* For the majority of samples being investigated at AMX using the focused fixed beam size of 7 µm × 5 µm, rastering (McPhillips *et al.*, 2002 ▸) is used to locate the sample(s) within a loop/mesh, locate the best diffracting volume(s) from the sample or simply determine whether or not a given condition gives detectable diffraction from an area as small as the beam size. For rastering, we write one master file for the entire raster and one data file per row or column depending on which is longer. All hdf5 files are written on the fast SSD dedicated buffer. For the spot finding application we use *Dozor* (Svensson *et al.*, 2015 ▸; Zander *et al.*, 2015 ▸) which can process hdf5 files directly. We are investigating the next generation of spot finder and rapid indexing algorithms. This will improve the upstream decision making for crystal centering in the automated data collection protocols.

*Data collection/*fast_dp_nsls2*.* Unless specified, all standard data collections with five or more consecutive degrees of rotation are automatically processed by the local implementation of *fast_dp* (Winter & McAuley, 2011 ▸). *Fast_dp* is a decision-making Python pipeline executing several instances of *XDS* for indexing, integration and scaling/merging using tools from CCP4 (Winn *et al.*, 2011 ▸) to generate merged reflection files and the associated log files. At the NSLS-II, we have modified *fast_dp* (*fast_dp_nsls2*) to run on a cluster with several nodes including one specialized node with overclocked CPUs (5 GHz for all six cores) for single-threaded applications. *Fast_dp_nsls2* uses the overclocked nodes for single processor steps to further speed up data reduction. *XDS* uses either *neggia* or *eiger2cbf* plugins to internally read the hdf5 data in a transparent way without the need to temporarily convert to cbf files. Additionally, we have added a function to *fast_dp_nsls2* that also outputs the most likely space groups within the point group using *pointless* and outputs the top three solutions as well as log files and a table. All codes are available or will be made available on gitHub channels (https://github.com/nsls-ii-mx). We have profiled our applications on AMD- and Intel-owned development clusters to optimize the CPU models in use. We have recently installed two AMD-based high-density compute servers (each with four nodes, each node with two CPUs with 32 cores each). This addition enables on-the-fly data processing relying on *autoPROC* (Vonrhein *et al.*, 2011 ▸) and will allow for the aforementioned fast-indexing algorithms to run in real time.

We have also written a new application called *fast_dp_pro* (unpublished), designed to optimize the longest possible sweep from the overall total oscillation range to maximize completeness, CC and minimize *R*_merge_. It sequentially maps shorter oscillation windows and generates the usual statistics that are further analyzed for automated selection of the best single sweep.

The two MX beamlines have access to a small, dedicated cluster made of 21 nodes comprising 716 cores (1432 threads). These nodes are connected to the GPFS fast storage (SSD buffers) and 1 PB disk-based GPFS arrays over a low-latency InfiniBand connection. Interconnect between nodes is over a 10 Gb ethernet. Additionally, each MX beamline has the dedicated workstation with overclocked Intel Core i7 CPU (six cores running at 5 GHz). We recently installed eight new high-density AMD nodes with a total of 512 cores (1024 threads) and 2 TB of RAM occupying only 4U space but generating about 3 kW of heat. These nodes were selected after benchmarking several of our MX pipelines, and this AMD CPU was selected because it delivered the fastest timing at a reasonable cost with limited IO bottleneck at the RAM to L3 Cache to CPU core.

All automated processes are launched by the data collection application (LSDC) using ssh calls, including distributed fast data reduction using *fast_dp_nsls2* for all data collection over five consecutive degrees (single collection as well as multiple automated data collections). Up to eight nodes are used for the rastering and a flexible number of nodes are used for the parallel steps of *fast_dp*. *Slurm* (Yoo *et al.*, 2003 ▸) is installed on all the compute nodes and can be used to distribute jobs from other beamlines, from the accelerator division, and also for specialized work when neither of the MX beamlines are in operation. *Slurm* services can be turned on and off by staff on pools of nodes or all. We find that this is the best way to use computing resources designed to cope with peak demand. We plan to implement post-processing of data using NSLS-II resources likely using *SynchWeb/ISPyB* (Fisher *et al.*, 2015 ▸; Delagenière *et al.*, 2011 ▸). All compute nodes and workstations are now running on RHEL 8.5 (Debian 8 or 9 until 2021).

### 2.4. Controls system and data collection application

**Figure 6:**
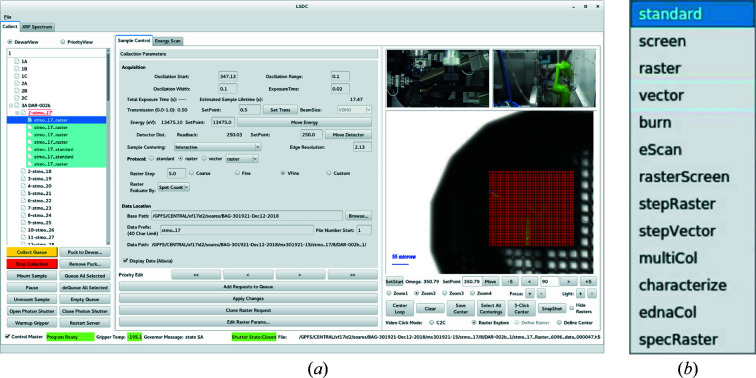
(*a*) The samples/requests view is displayed in the left column, data acquisition parameters including access to all protocols are set in the center, and the sample video view including centering modes and interactive feedback of the raster heat map is displayed in the right column. Two of the pan zoom tilt (PZT) camera views are also displayed in the GUI so that users have a good overview of what is happening in real time at the beamline. (*b*) The drop-down menu of the currently available protocols.

The detailed description of the controls systems in use at the AMX and FMX beamlines is given elsewhere (Schneider *et al.*, 2021 ▸). The NSLS-II standard applications in use at the AMX beamline are: *EPICS*, *Control System Studio* (*CSS*), *Bluesky* and *Jupyter Notebook*. The inhouse main data collection environment and graphical user interface called LSDC (Life Science Data Collection) was developed inhouse for the AMX and FMX beamlines so that users interact with a single GUI to perform data collection (Fig. 6 ▸). It has recently been extended to the NYX beamline, so that now all NSLS-II MX beamlines present an identical interface to the users. Additionally, we have installed *ISPyB* (Delagenière *et al.*, 2011 ▸) and *SynchWeb* (Fisher *et al.*, 2015 ▸) for users and staff to monitor data collection and data processing statistics in real time.

#### 2.4.1. LSDC

The LSDC application is designed to enable a variety of experiments with a wide range of complexity by implementing several crystal centering methods and data collection protocols. The three most commonly used protocols are the standard, raster and vector protocols.

The standard protocol is the standard rotation method (Arndt & Wonacott, 1977 ▸) that performs a continuous rotation of the crystal in which users select the total oscillation range and other parameters including transmission, exposure time, detector distance and oscillation width per frame.

The raster protocol allows users to rapidly interrogate a sample by evaluating the diffraction patterns from a chosen sample area. Users select the corners of an area to be mapped by the beam with a given step size (20, 10, 5 or custom; in µm). Defined raster areas are mapped to a rectangular grid that encompasses the defined area in which each row (or column) of the grid corresponds to a separate vector scan. This configuration allows for a single arming of the Eiger X 9M, and for parallelization of the spotfinder processing, resulting in a considerable speedup. For instance, a 200 µm × 200 µm area can be rastered with a 10 µm step size and 2 s of total exposure time.

The vector protocol, also known as helical scanning, allows users to select a start and an end position so that the sample is not only continuously rotated but also translated following the linear trajectory of the defined vector. In the vector protocol, users can expose the sample to a higher flux by distributing the radiation dose over much larger volumes. Users typically perform two orthogonal rasters and, after inspection of the corresponding heat maps and diffraction patterns (using the Dectris Albula diffraction viewer), center the samples at the two end points.

For experiments involving many small crystals (loaded onto a loop or a mesh) we have developed the *stepRaster* protocol in which a pre-set oscillation range is collected at each voxel of the raster, and, if five continuous degrees or more are collected, *fast_dp_nsls2* is launched to analyze the data. This protocol enables automated data collection on high sample volumes, for example to study protein dynamics from 100 s to 1000 s of crystals (Soares *et al.*, 2022 ▸). To help users determine early in their experiment whether or not crystals diffract they can use *rasterScreen*. In this protocol, the sample pin is mounted, *autoLoop* centering (*XREC*; Pothineni *et al.*, 2006 ▸) is used and an area larger than the loop is automatically set for a raster using pre-set parameters [step size, transmission, exposure time, oscillation width (0–0.3°)], and data are analyzed using *Dozor*. At completion of all *rasterScreen* collections, heat maps are saved and made available for user interaction.

With the beam size and flux of AMX, it is key to make users aware of radiation damage. We have a simple application for estimating the expected lifetime of a crystal comprising an average protein with physical dimensions matching the size of the beam; lifetime is then estimated given the transmission and the energy of the beam. However, to further refine the expected lifetime of a crystal, we have developed the *burnProtocol*; in this protocol, after a sample is centered (*interactive*, *autoLoop* or *autoRaster*), it remains still (no rotation so that during the protocol no fresh volume is being brought into the beam). The sample is then exposed for 2 s at 200 frames s^−1^ to the full beam, or an estimated dose of 70 MGy. The *burnProtocol* experimentally measures radiation sensitivity for a given macromolecular crystal in which results are reported as an absolute sample lifetime (in seconds at a given transmission or dose in MGy) that is for an area of the sample the size of the beam. The majority of the samples at AMX are larger than the beam size and the absolute calculated lifetime from the *burnProtocol* can be fed to *RADDOSE-3D* (Zeldin *et al.*, 2013 ▸) to empirically estimate lifetime based on crude sample size measurements. These steps will be automated in the future.

Other protocols available at the AMX and FMX beamlines are: *stepVector* (collect a fraction of the total rotation at one location, translate the sample further away on the trajectory of the vector, collect the next continuous rotation until collection is complete); *characterize* (collect two frames, at 0 and 90° with 0.5° oscillation range, and run the *EDNA* characterization); *ednaCol* (run the *characterize* protocol and then collect the single data set following parameters determined by *EDNA*); *multiCol* (perform a raster and then collect pre-determined data sets on each area meeting the required settings: often users select a resolution as threshold parameter); and *specRaster* (for some specialized experiments, collect a fluorescence spectrum at each area of the raster). The underlying application and architecture is such that complex new protocols can be developed and then implemented at the GUI level. The total amount of time required to run the *EDNA* *characterize* protocol (collection of the two frames and calculation) is 15 s. The calculation runs on the dedicated 5 GHz overclocked workstation.

#### 2.4.2. Crystal centering

The LSDC application contains four methods to center a sample: *click to center* (*C2C*), *3-Click Center*, *CenterLoop* and *autoRaster*. The default mode is *C2C*, where users center the sample in the cross hair (rotation axis and beam center) interactively with a left click of the mouse; this step is repeated until the crystal is satisfactorily centered. The *3-Click Center* is a semi-automated self-guiding workflow where the Ω axis is sequentially rotated at three strategic values and users click on the center of mass of the sample at each of these three angles. The *CenterLoop* function executes the *XREC* (Pothineni *et al.*, 2006 ▸) application and captures nine images (every 40°); the center of mass of the loop and ‘face on’ absolute orientation are calculated and motions are executed. The *autoRaster* centering mode was developed for the first fully automated data collection mode, that is described below. In the *autoRaster* centering mode, the SPINE base is mounted and the *CenterLoop* application is run twice; this repetition is to ensure that shorter or longer loops are given a chance to be centered; the loop ends up in ‘face on’ orientation; then a rectangular area is defined with a fixed size of 630 µm × 390 µm (with coarse 30 µm × 30 µm step size), this initial fixed total area was optimized based on the distribution of sample sizes AMX and FMX observed during the first year of operation; at completion of the raster, the crystal is then centered based on a score derived from the *spotFinder* application; this initial coarse centering is followed by a smaller 90 µm × 90 µm raster (with a finer grid size of 10 µm × 10 µm); the sample is centered again on the voxel that has the maximum signal, *e.g.* number of reflections; the loop is then rotated by 90° so that the loop is now ‘parallel’ to the beam and a 290 µm vertical line scan (with 10 µm step) is executed and *spotFinder* analysis step ensures that the sample is centered in all orientations. Rastering parameters (exposure time, transmission, distance, oscillation range per frame, *etc.*) are stored separately for rasters and they may differ from parameters for the standard and vector protocols.

#### 2.4.3. Automated data collection

For automated data collection on multiple samples, we developed a queuing collection mode that will perform pre-determined collections strategies on samples that are selected for each given collection. Multiple collections can be set, and multiple queues as well. The simplest implementation is optimized for large samples (smallest dimension greater than 40 µm) of well studied crystals, which is generally the case for ligand binding or fragment screening for drug development projects; however, the protocol works well for any uniform crystal 40 µm long or more. The protocol includes the following steps: the sample is mounted on the goniometer; there is a pre-set wait time (about 15 s) to allow the ice between the base and the magnet to fully melt so that the sample will not drift during the following steps; then the sample is centered either using the *centerLoop* (provided that the loop size matches the crystal size) or *autoRaster* mode (described above); data are then collected in the standard protocol with the settings selected, *e.g.* starting angle, total oscillation, detector distance; if users have selected *autoProcess*, data will be automatically reduced and in the case of ligand binding studies the *dimple_nsls2* pipeline is subsequently executed, given that users provide a PDB model file. At the end the application unmounts that sample and mounts the next sample in the queue and continues. Currently, AMX can achieve 18 samples per hour with the *autoRaster* mode (X-ray used for sample centering) and 25 samples per hour if using the *CenterLoop* mode. The default total oscillation range is 180° but users can select *ednaCol* in the list of protocols available on the drop-down menu and, in this case, the *EDNA characterize* calculation results are used for immediate data collection on each sample; in this mode, to be fully compliant with the indexing/strategy calculation requirements, two frames 90° apart with 0.5° oscillation per frame are collected at a short distance and analyzed.

We are currently developing a simple *autoVector* data collection protocol that relies on two orthogonal large area rasters and optimization of the longest vector.

## 3. Operation and current status

### 3.1. Access modes

There are three current ways users can access the beamline – local, remote and automated – and each is described below.

*Local* mode is where users travel to AMX (NSLS-II, BNL, Upon, NY, USA) and collect data using beamline workstations a few steps away from the experimental hutch. This mode is optimal because of the direct interactions between users and expert staff benefiting both parties; we have observed that most users are more productive and efficiently trained that way. Staff members benefit from these direct interactions as they can plan future improvements based on requirements that are initiated by users to further improve their scientific advancement.

Following at least two local visits to either AMX or FMX, users are then qualified to use *Remote* access. This is to ensure users have been exposed to most functionalities and interacted with staff so that they are aware of the capabilities of the beamlines and have experimented with various data collection methods multiple times. Users connect to a beamline workstation using the NoMachine NX native application (NX client) and dual factor authentication.

Users connect to one of the beamline virtual terminal servers using NX Client and operate the beamline using *lsdcGui* and *Albula* as the main user interfaces. Users benefit from our *SynchWeb/ISPyB* implementation and can monitor data collection and data analysis statistics near real time. All data are transferred to the user home institution using *Globus* (Chard *et al.*, 2016 ▸). It is highly recommended to use a monitor with at least QHD resolution (>2560 × 1440), but for more challenging experiments best user experience is achieved with two QHD monitors. All communications between beamline personnel and users is done using a chat application. *Slack* is currently being implemented at the beamlines to support real-time communication during beam time between the beamline staff and the user groups.

Fully *automated* access is the mode where users send samples and beamline staff scientists perform the automated data collection based on user requirements. Data access and *ISPyB* links are shared with the user(s) at the beginning of each beam time so that they can download their data and monitor feedback from data processing. This mode is useful as it brings some flexibility to the operation of the beamline. Delayed deliveries or beam time cancellations are rare events, but they can disrupt beamline operations; unscheduled automated access allows for data collection on samples meeting the specified requirements (SPINE base with nylon loop and a single crystal with the smallest dimension greater than 40 µm) and is increasingly used to ensure data are collected on all samples. It is also used to accommodate unplanned influxes of samples.

Finally, we are planning to implement a hybrid access mode where users will interact with *SynchWeb/ISPyB* to set up data collection and monitor data processing results to possibly reload samples in the queue.

### 3.2. COVID-19 updates

The AMX and FMX beamlines were in continuous operation during the peak phase of the COVID-19 pandemic in spring of 2020, extending operation beyond what was originally planned so that academic and industrial users had access to valuable resources for therapeutics development treating directly and indirectly the infection caused by the SARS-CoV-2 virus (Jakoncic *et al.*, 2020 ▸). During the pandemic, new users were remotely trained by staff over a 2 h-long period of dedicated beam time with test samples provided by the beamline staff and using video conferencing tools. This training session is required to ensure that new users are sufficiently trained and confidently able to perform the most common experiments and be more productive so that their scientific challenging questions are best answered.

### 3.3. Proprietary access

At the two MX beamlines, we allocate a small fraction of the available beam time so that mostly US-based pharmaceutical companies can access state-of-the-art instruments to support their R&D efforts. Most of these pharmaceutical users profit from the fully automated data collection but certainly benefit from the advanced tools and beam properties to tackle their most challenging and pressing projects as well. Since we share a common goal of very high throughput with high efficiency, these users are a good fit for the beamline and continuous improvement in beamline performance enabling increased productivity are designed with them in mind too.

### 3.4. SynchWeb/ISPyB

The *ISPyB* (Delagenière *et al.*, 2011 ▸) database (implemented in a *MariaDB* instance) and *SynchWeb* (Fisher *et al.*, 2015 ▸) application were installed on Debian computers on the NSLS-II Controls network; *SynchWeb* is available to users while they are logged into the computers for remote experiments. The *ISPyB* schema has been implemented from a Github-based codebase (https://github.com/DiamondLightSource/ispyb-database). An upgrade from Debian to Red Hat Linux Enterprise is in progress. LSDC stores information in the *ISPyB* database using the *ISPyB-API* Python library (https://pypi.org/project/ispyb/). Currently, the overall dataset spotfinding plot, standard and vector dataset processing results from *fast_dp*, and rastering results are available to the user. Future work is planned to provide dataset postprocessing abilities, and sample shipment handling.

## 4. Example of experiments carried out at the AMX beamline and productivity

### 4.1. Techniques to leverage mini-beams for ligand discovery

**Figure 7:**
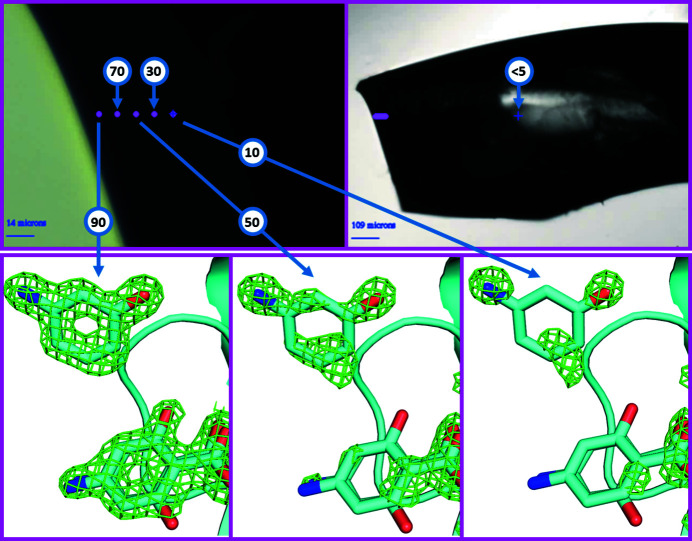
Diffraction data were obtained from one large lysozyme crystal soaked with 50 m*M* 4-amino­salicylic acid (PAS) and 50 m*M* 3-amino­phenol (3AP) for 15 min prior to cryo-cooling. Diffraction data were obtained from five regions located near the crystal surface, in 10 µm shells (top left). Diffraction data were also obtained from the center of the crystal (top right). The observed occupancy from the near-surface shells was 90%, 70%, 50%, 30% and 10%, respectively (bottom). The observed occupancy from the center of the crystal was near zero (<5%).

Bound ligands can be observed either soaked into protein crystals or co-crystallized with protein crystals. Soaking is generally preferred because it is easy to integrate into time- and cost-efficient workflows, though co-crystallization can yield binding poses that are less sterically constrained and hence more native-like (Wienen–Schmidt *et al.*, 2021 ▸). Even though soaking is an efficient method for high-throughput screening of potential ligands, it can fail in cases where the ligand does not diffuse sufficiently into the crystal lattice or where the protein-ligand co-crystal is not stable. In both cases, the time during which useful soaking occurs is less than the time needed for the ligand to fully penetrate the crystal lattice (either because ligand diffusion through the crystal lattice is impractically slow or because the ligand is not stable in the crystal). In cases where the ligand penetrates only slightly into the crystal, it may still be detected if the X-ray beam is finer than the penetration depth of the ligand. We have used the 7 µm × 5 µm beam at AMX to detect ligands bound with high occupancy near the surface of protein crystals, even though the occupancy of the ligand within the entire volume of the crystal is near zero. The strategy is to orient the sample so that diffraction originates only from the surface layer that has been penetrated by the ligand. We soaked lysozyme crystals with a 50 m*M* equimolar solution of 4-amino­salicylic acid (PAS, a known unstable lysozyme ligand) and its degradation product 3-amino­phenol (3AP). Both PAS and 3AP are observed to soak slowly into lysozyme crystals, requiring approximately 100 s for each micrometre of crystal depth to attain full occupancy (data not shown). After 15 min, the crystal was cryo-cooled and diffraction data were measured from five shells each sequentially 10 µm deeper into the crystal lattice (Fig. 7 ▸). The occupancies of both ligands were then determined using standard crystallographic methods.

### 4.2. Identify individual polymorphs using diffraction from dynamic proteins

Small differences are often observed in diffraction data measured from different crystals of a single protein (these differences may originate either from small differences in the micro-environment of each crystal or from natural dynamism in the molecule in solution). Since an X-ray micro-beam illuminates a small region that is likely to be homogeneous, we used 146 diffraction data sets obtained from crystals of chymotrypsinogen (ChTg) using the AMX micro-beam to develop new methods to accurately cluster diffraction data sets using a novel two-factor clustering method (Nguyen *et al.*, 2022 ▸; fully automated data were measured from 511 samples, of which 146 processed to high resolution in the correct space group). The protein is a single chain of 245 residues, of which four residues (147–150) were not resolved.

**Figure 8:**
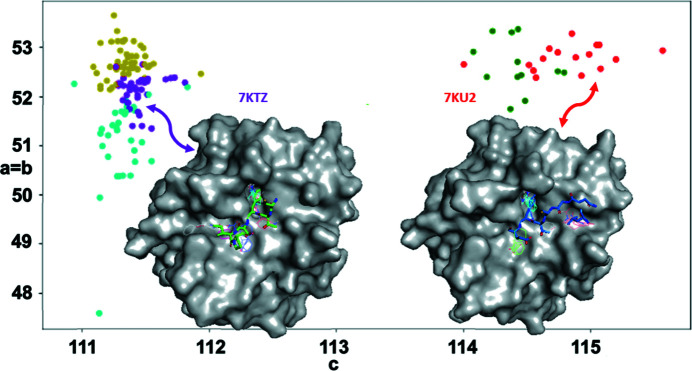
Diffraction data from 146 ChTg crystals. Each data set is shown as a dot according to its unit cell values (*x* and *y* coordinates) and according to its grouping after to two-factor clustering (five colors). The data readily partition into two main groups using conventional one-factor clustering (two main populations at left and right), and further subdivide into five sub-groups using two-factor clustering. *FTMap* was used to compare the predicted binding properties of the different structural polymorphs, which differed markedly between the two main cluster groups (the inset shows binding poses superposed on the protein envelopes of 7ktz and 7ku2).

We obtained our initial structure, PDB entry 7kty, from a merge of all 146 data sets and called this the average structure. We used *REFMAC* and *Coot* to refine the structure and reduce the *R* value to about 18%. Cell-based clustering was used to filter the diffraction images to be ones from a single point group. Then structure-factor-based clustering was applied, yielding clusters organized into a dendrogram. The average structure corresponds to the top cluster from the hierarchical cluster analysis. Data clusters further down in the dendrogram revealed several intriguing polymorphs of ChTg. The data readily grouped into two broad categories using conventional single-factor clustering methods but could be further partitioned into five sub-categories using two-factor clustering (Fig. 7 ▸, circular data points with different colors). One of the ways in which these five polymorphs differed was their predicted binding properties using the *FTMap* software (Fig. 8 ▸ illustrates the highest affinity poses for polymorphs 7ktz and 7ku2; see Kozakov *et al.*, 2015 ▸).

### 4.3. Room-temperature collection

A small number of MX experiments are performed at room temperature (RT) and only a few of these are seen at AMX. Data collection from samples at RT are done in either short capillaries, or with loops embedded in paratone oil so that buffer solution does not evaporate and damage the sample during the transfer or in a commercial sleeve performing standard data collection using the rotation method. Data are also collected from *in situ* compact crystallization trays (Soares *et al.*, 2021 ▸). This method uses acoustic droplet ejection to set up the hundreds of crystallization conditions. All samples for RT data collection are loaded to the goniometer by hand and for single-crystal data collection it is crucial to attenuate the beam as low as 0.1% so that samples are not obliterated in a few frames. We have collected RT data sets on the main protease from the SARS-CoV-2 in the presence of several antiviral potential drug targets (article in preparation); for this purpose, the beam size was expanded to 25 µm × 25 µm so that complete data sets could be collected from single crystals to a resolution of 2.7 Å while exposing the crystals to reduced radiation dose.

**Figure 9:**
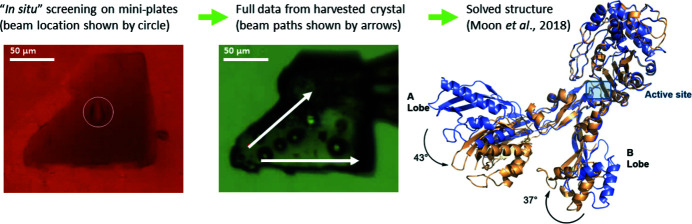
Workflow for *in situ* screening on mini-plates followed by full data collection on a harvested crystal. A single crystal was obtained from acoustically prepared nanolitre crystallization screens. The crystal was screened *in situ* at room temperature (left), then full vector data were obtained at 100 K after harvesting (middle), and the data were used to solve the structure (right, purple structure; see Moon *et al.*, 2018 ▸).

Data collection from samples in the *in situ* trays can be performed with the small beam at the highest frame rate of the detector for probing whole crystallization drops. Because of physical dimensions and space around the sample area, these *in situ* crystal trays can only be rotated ±5° from the vertical orientation so that collisions with the collimator can be avoided; additionally, data collection from these trays is done with the cryo-stream retracted to increase the available envelope. The workflow for these can be: screening all the drops from one compact tray and execution of partial data sets on all ‘found crystals’ with the nominal AMX beam size or with the increased beam size (25 µm × 25 µm) so that mapping drops is not only faster but ‘non-destructive’ with low transmission. One such tray was tested at AMX during the scientific commissioning of the beamline (Moon *et al.*, 2018 ▸) with BR5 samples; at the time we operated the beamline with minimal hardware and the best sample detected from the drop screening at AMX was collected and flash cooled for single-crystal data collection at 100 K (Fig. 9 ▸).

We are currently working on developing workflows for automated operation of single *in situ* trays following workflow from the *multiCol* protocol.

### 4.4. Current status and statistics

**Figure 10:**
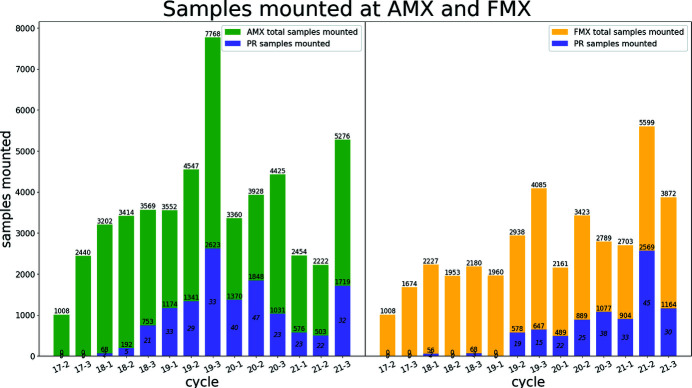
Sample throughput at the AMX and FMX beamlines. Total number of samples mounted at each of the two MX beamlines at the NSLS-II including samples from proprietary users. Sample throughput in calendar year 2020 is lower than expected due to the effect of the pandemic. Nevertheless, the AMX and FMX beamlines remained in operation to support ongoing efforts to deal with the pandemic including inhouse research on the main protease, the receptor binding domain of the spike protein and a peptide preventing fusion of the SARS-CoV-2 with the host cell membrane.

At AMX, we provide 75%, or more, of the available time with beam for user operation including general user (GU), block allocation groups (BAG), rapid access (RA), proprietary research (PR) as well as a small fraction of time to be used for developmental projects that will enhance the scientific portfolio of the beamline including data collection from 100 s to 1000 s of samples to investigate protein dynamics. The rest of the available time is used for technical commissioning including starting up the beamline after each long shutdown and implementing planned new capabilities. The NSLS-II accelerator, when in user operation, operates 24/7 with three eight-hour shifts per day. To maintain continuous operation of the beamline we have trained the floor coordinators of the NSLS-II so that they can provide robotics support in rare cases of hang up. They are trained to respond to local users, remote users and ongoing efforts are made so that they can also recover from fully automated data collection during the unstaffed nights. During the COVID-19 pandemic, all proposals related to the pandemic were given rapid access time. The total number of user samples, per beamline and per cycle, is shown in Fig. 10 ▸.

### 4.5. Medium-term planned improvements

Ongoing development of a helium flight path between the sample and the detector surface at the FMX beamline will be deployed at AMX to increase signal to noise at energies lower than 7 keV. The recent implementation of *Dozor* as the main spotfinder application together with the plan to implement two orthogonal large raster maps with 10 µm × 10 µm raster step size will enable automated data collection using the vector protocol for samples with a smallest dimension of 20 µm. This will be followed by automated multiple data collection from either a large sample or multiple small samples in a loop/mesh/mount. We are also implementing more automated data processing including multiple data reduction pipelines and hierarchical cluster analysis (HCA) to assemble the best data depending on the type of experiment in progress as well as HCA to study protein dynamics (Nguyen *et al.*, 2022 ▸). Finally, we are preparing for increased demand beyond what AMX and FMX can currently support during the Advanced Photon Source upgrade period. We are implementing tools and optimizing the application to achieve shorter down times and increase achievable throughput.

## 5. Conclusion

AMX saw first light in March 2016 – the first diffraction pattern and complete data collection from a test sample in July 2016. AMX started general user operation in February 2017 after several scientific commissioning experiments scheduled in fall 2016. The number of users, samples and also reliability have continuously improved over the last five years thanks to the hard work of the CBMS staff and the user community. At AMX, we are currently implementing all necessary hardware tools and software so that automated data collection from the majority of samples can be performed, preferably during the evening hours and the weekends so that users/staff can interactively perform experiments during the daytime for the most challenging samples necessitating expertise unavailable at the moment from the application point of view. We are also planning to develop machine-learning tools to enable autonomous operation of the beamline and this will be communicated once achieved. We are aiming to deliver a throughput of 1000 samples per day for specialized experiments including fragment screening, ligand binding and protein dynamics from many hundreds of structures.

## Figures and Tables

**Table 1:** AMX key performance parameters – specifications of the AMX photon delivery system and achievable parameters

Energy range	5–18 keV
Wavelength range	0.7–2.5 Å
Flux at focus†	4.3 × 10^12^ photons s^−1^
Focal spot	7 µm × 5 µm
Divergence	1 mrad × 0.35 mrad
Achievable resolution‡	0.8–3 Å

† At 13.475 keV (Fig. 2 ▸).

‡ At the minimum detector distance of 100 mm and at the two extreme energies (18 and 5 keV).
